# Determining factors of functioning in hemodialysis patients using the international classification of functioning, disability and health

**DOI:** 10.1186/s12882-022-02719-5

**Published:** 2022-03-24

**Authors:** Luciana M. M. Santos, Pedro Henrique S. Figueiredo, Ana C. R. Silva, Patrícia C. Campos, Gabriele T. Gonçalves, Jaqueline de Paula C Freitas, Fidelis Antônio da Silva Junior, Jousielle Márcia Santos, Frederico L. Alves, Vanessa G. B. Rodrigues, Emílio Henrique B. Maciel, Maria Cecília S. M. Prates, Borja Sañudo, Redha Taiar, Mario Bernardo-Filho, Vanessa P. Lima, Henrique S. Costa, Vanessa A. Mendonça, Ana Cristina R. Lacerda

**Affiliations:** 1 Centro Integrado de Pós-Graduação e Pesquisa em Saúde (CIPq-Saúde), Universidade Federal dos Vales do Jequitinhonha e Mucuri, Diamantina, Brazil; 2 Laboratório de Fisiologia do Exercício – LAFIEX – CIPq/Saúde, Universidade Federal dos Vales do Jequitinhonha e Mucuri, Diamantina, Brazil; 3 Universidade Federal dos Vales do Jequitinhonha e Mucuri, Rodovia MGT 367 - Km 583, n 5000, Alto da Jacuba, Diamantina, Minas Gerais 39100-000 Brazil; 4 Programa de Pós-Graduação em Reabilitação e Desempenho Funcional, Universidade Federal dos Vales do Jequitinhonha e Mucuri, Diamantina, Brazil; 5 Laboratório de Inflamação e Metabolismo – LIM – CIPq/Saúde, Universidade Federal dos Vales do Jequitinhonha e Mucuri, Diamantina, Brazil; 6 Faculdade de Medicina, Universidade Federal dos Vales do Jequitinhonha e Mucuri, Diamantina, Brazil; 7 Unidade de Hemodiálise do Hospital Santa Casa de Caridade de Diamantina, Diamantina, Brazil; 8 Department of Physical Education and Sports, Universidad de Sevilla, Seville, Spain; 9 MATériaux et Ingénierie Mécanique (MATIM), Université de Reims Champagne-Ardenne, Reims, France; 10 Mechanical Vibration Laboratory and Integrative Practices (LAVIMPI), Biophysics and Biometrics Department, Institute of Biology Roberto Alcântara Gomes and Piquet Carneiro Polyclinic, Universidade do Estado do Rio de Janeiro, Rio de Janeiro, Brazil

**Keywords:** Hemodialysis, Biomarker, Body composition, Parathormone, Alkaline phosphatase, Physical activity level

## Abstract

**Background:**

Hemodialysis (HD) treatment affects functioning, physical activity level, clinical biomarkers, and body composition. However, the association between these variables with functioning, considering International Classification of Functioning, Disability and Health (ICF) domains remains unclear. Thus, the aim of this study was to investigate the possible association between physical activity, biomarkers, and body composition with functioning in HD patients in reference to the ICF.

**Methods:**

Eighty HD patients performed different tests grouped according to ICF domain: Body structure and function – handgrip strength (HS), 5-repetition sit-to-stand test, and 60-s sit-to-stand test (5-STS, 60-STS, respectively); Activity – short physical performance battery (SPPB); and Participation – participation scale questionnaire. Physical activity [Human Activity Profile questionnaire (HAP)], body composition (Dual-energy X-ray absorptiometry), Parathormone (PTH), and alkaline phosphatase were analyzed as possible variables associated with ICF domains. Data analyses were performed using simple and multiple regression models adjusted for age, duration of HD, and diuresis volume.

**Results:**

In the body structure and function domain, appendicular lean mass, PTH level, and age were associated with HS (R^2^ = 0.558); HAP and PTH were associated with 5-STS (R^2^ = 0.263); and HAP, PTH, duration of HD, and age were associated with 60-STS (R^2^ = 0.337). In the activity domain, HAP, PTH, alkaline phosphatase, duration of HD, age, and body fat were associated with SPPB (R^2^ = 0.689). Finally, only HAP was associated with the participation scale (R^2^ = 0.067).

**Conclusion:**

Physical activity and PTH levels are determinant protagonists of functioning in all ICF domains in hemodialysis patients.

**Supplementary Information:**

The online version contains supplementary material available at 10.1186/s12882-022-02719-5.

## Background

End Stage Renal Disease (ESRD) is a worldwide public health problem; the prevalence in the Brazilian population in 2018 was 640 patients per million of the population, and the cost of treatment with hemodialysis (HD) was reported to be around 2.2 billion reais per year [1].

Despite HD treatment being crucial for the management of symptoms and increasing survival in ESRD, it also causes numerous deleterious effects [2–6]. As a result of HD, non-specific inflammatory processes, metabolic acidosis, abnormalities in vitamin D metabolism and/or serum calcium and phosphorus levels, and, consequently, hyperparathyroidism are known to occur [2]. Furthermore, reductions in lean mass and bone mineral density are also frequent in this population [3]. These events related to HD are associated with a loss of functionality, a high prevalence of frailty, and an increased risk of mortality [4]. In addition, there is a decrease in performance in activities of daily living and independence [5], which may compromise the social participation and quality of life of HD patients [6].

The International Classification of Functioning, Disability, and Health (ICF) is widely used to evaluate different domains of functioning, including body structure and function, activities, and social participation, in many chronic conditions worldwide [7, 8]. Thus, HD treatment may severely affect different physical and psychosocial aspects, contributing to a decline in functioning [9]. Accordingly, healthcare staff must precisely identify the clinical and functional problems of HD patients in regard to the ICF^9^. Previous studies have demonstrated impairments in balance, overall muscle strength (including respiratory muscle strength), and cardiorespiratory fitness in HD patients [10–12]. However, specific evaluation of the body structure and functions can provide an incomplete functional evaluation [13] because the activities and social participation of HD patients might be also impaired, possibly contributing to reduced functionality and poor survival [14, 15].

Although a previous study investigated associations between clinical and functional aspects in HD patients [16], due to the complexity of the disease and its multiple repercussions on the overall health of patients, there remains a gap in the identification of possible clinical variables (e.g., body composition, physical activity level, biomarkers) associated with functioning in reference to the ICF in a complementary and integrated view in HD patients. Therefore, the present study aimed to investigate possible variables associated with functioning in reference to ICF domains in HD patients. Thus, it was hypothesized that body composition, biomarkers such as parathormone, alkaline phosphatase, vitamin D and C-reactive protein, and regular physical activity would be determinants of functioning in HD patients.

## Methods

### Study design

This was an exploratory, cross-sectional study conducted between August and December 2019 in the HD unit of the Hospital Santa Casa de Caridade of Diamantina. The study was approved by the Human Ethics Committee of the Universidade Federal dos Vales do Jequitinhonha e Mucuri (Protocol = 3.612.157), and carried out in accordance with the declaration of Helsinki (2013). All the HD patients provided written informed consent to participate in the study.

The study included patients over 18 years old, with ESRD, on HD treatment three times a week for at least 6 months, and with arteriovenous fistula for HD access. Exclusion criteria were HD patients with any contraindications to performing exercise tests or practicing physical exercise. Patients using corticosteroids or non-steroidal anti-inflammatory drugs were also excluded.

GPower software, version 3.1.9.2, was used for sample size calculation, which was estimated based on a pilot study with 10 hemodialysis patients. Using all dependent variables, inputting an effect size of 0.67 (for 60 s sit-to-stand test, i.e., the lowest obtained), 14 possible predictors, probability of error set at 1%, and a power of 99%, a total of 80 patients was estimated.

### Outcomes

Handgrip strength (HS): According to the American Society of Hand Therapists (ASHT), the assessment was performed using the Jamar® dynamometer (Asimow Engineering Co, Los Angeles. CA), adjusted to the second position [17]. Volunteers remained in a seated position, with the shoulder in adduction and 90° at the elbow joint, with the forearm in a neutral position [18–20]. Three measures of HS of the arm without arteriovenous fistula were performed and the average of these measures was used for analysis. An interval of 1 min was given between each measurement [21].

5-repetition and 60-s sit-to-stand tests (5-STS and 60-STS*):* The volunteers started in a sitting position, with their arms crossed over their chest and their back against the chair. The seat was at a height of approximately 43 cm. The researcher was positioned next to the volunteer, giving instructions and preventing a fall. For the 5-repetition sit-to-stand test, the time to complete the 5 repetitions was registered using a digital timer [22]. For the 60-s sit-to-stand test, the number of repetitions during 60 s was counted and noted [21].

Short physical performance battery (SPPB): The instrument consists of the following tests: static standing balance, walking speed at a normal pace for 4 m, and muscular strength of the lower limbs estimated by the sit-to-stand test without the aid of the arms five times [21, 23]. For each test, the performance was obtained by a score from 0 (worst performance) to 4 (best performance), and the scores were then added together to calculate the final points, resulting in a maximum score of 12 points. A score ≤ 8 points indicates poor physical functioning [24].

Participation scale: This instrument was translated and adapted for the Brazilian population and is based on an interview covering 18 items to measure problems perceived in the main domains of participation [25]. Subjects with a score greater than 12 were classified as having participation restrictions [25, 26].

Human Activity Profile (HAP): HD patients were asked to complete a validated and cross-cultural adapted questionnaire related to physical activity level [27, 28]. The HAP is a scale designed to survey the execution of common physical activities and consists of 94 items ranked in ascending order of energy requirements [29]. The adjusted activity score was recorded and higher scores indicate higher activity levels [29].

Dual-energy X-ray absorptiometry: A densitometer (Lunar Radiation Corporation, Madison, Wisconsin, USA, model DPX) was used to determine total body mass, body fat, appendicular lean mass (ALM), and bone mineral density (BMD) [30]. ALM was obtained as the sum of the muscle mass of the four members. Moreover, the total and lumbar spine (L2-L4) BMD (g/cm^3^) were also assessed [28].

Biomarkers: Two 10 ml tubes of blood samples without anticoagulant were collected from each patient for the analysis of 25-hydroxyvitamin D (vitamin D), PTH, and CPR. CPR was measured using the turbidimetric method (Biotéctica Indústria e Comércio, Varginha, MG, Brazil) [31]. PTH was determined through electrochemiluminescence (Modular Analytics E170, Roche, Mannheim, Germany). Vitamin D levels were measured using a direct competitive chemiluminescence immunoassay (Architect i2000, Abbott, IL, USA). The observed reference range was 9.3–47.9 ng/mL [32]. The lowest reported value was 4 ng/mL, and the interassay coefficient of variation (CV) was < 20% [33].

### Procedure

Preliminary session: The anamnesis of the HD patients, including personal and disease-related factors (gender, age, comorbidities, duration of HD, diuresis volume, fractional urea clearance - Kt/V, serum levels of hemoglobin - Hb, alkaline phosphatase, and ferritin) were obtained from regular medical records. Subsequently, HD patients were familiarized with all physical functioning tests and with the questionnaires [HAP and the Participation Scale] to be completed in the following session.

The first experimental session: Immediately before the first hemodialysis session of the following week, the HS of the HD patients was assessed. Then, during the hemodialysis session, the HAP and the participation scale questionnaires were applied to assess physical activity level and social participation, respectively.

The second experimental session**:** Immediately before the second hemodialysis session, physical performance tests were performed: SPPB, 5-STS, and 60-STS. The blood sample was collected during puncture of HD patients’ arteriovenous fistula for later analysis of biomarkers. Finally, after the end of the hemodialysis session, body composition was assessed using DXA. In addition, weight and height were measured to calculate the body mass index (BMI).

The reliability of all clinical-functional tests was greater than 80% for all tests [21, 29, 34, 35]. All dependent outcomes were grouped according to ICF [7] domains as follows: **(1)** Body structure and function – upper limb strength: HS; lower limb strength: 5-STS; and endurance: 60-STS; **(2)** Activity – SPPB; and **(3)** Social participation – participation scale; and their association with independent outcomes (age, duration of HD, diuresis volume, BMI, body fat, ALM, BMD total, BMD spine, BMD hip, D vitamin, PTH, CRP, Kt/V, Hb, alkaline phosphatase, ferritin, HAP) was subsequently investigated.

### Statistical analysis

Data analyses were performed using SPSS version 22.0 (SPSS Inc., Chicago, IL, USA) (Additional file 1). The sample distribution and homoscedasticity were assessed using the Shapiro Wilk test and the Levene test. The continuous data were expressed as mean and 95% confidence interval, and categorical variables as absolute number and percentage. Confirmatory analyses of the association, using simple linear regression followed by stepwise multiple linear regression models were performed with independent variables that presented *p* < 0.20 in the correlation tests (Pearson or Spearman test) (Additional file 2) and *p* < 0.10 in simple linear regression (Additional file 3). The significance level was set at 5%. The models were adjusted for age, fractional urea clearance, duration of HD, and diuresis volume. To perform the multiple regression analysis, four assumptions were adopted: linearity, distribution of residuals, homoscedasticity, and the absence of multicollinearity. The linearity of the independent variables and residuals was checked by scatter plots and the distribution of residuals was analyzed using the histogram. The homoscedasticity was verified by the scatter plot and characterized by the residuals equally distributed on the regression line. The absence of multicollinearity was defined as variance inflation factor (VIF) values below 10.0. In addition, the auto-correlation of the variables was verified through the Durbin-Watson test and values between 1.5 and 2.5 showed no autocorrelation in the data.

## Results

**Fig. 1:**
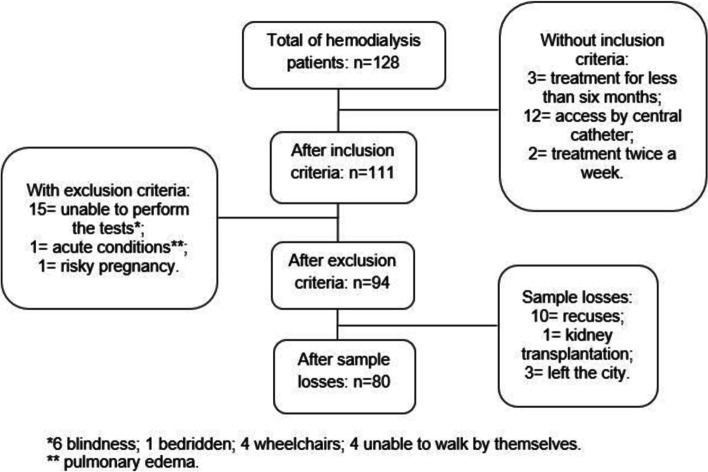
Flowchart of inclusion and exclusion criteria

After applying the inclusion and exclusion criteria (Fig. 1), ninety-four HD patients were eligible for the study, but with sample losses a total of eighty participated (61% men; age 20 to 90 years, mean 53 ± 16) (Table 1). Systemic arterial hypertension was the most prevalent etiology of ESRD, and dialysis data on fractional urea clearance (kt/v indexes) demonstrated the efficiency of HD treatment according to the National Kidney Foundation [36] (Table 1). The physical functioning characterization is presented in Table 2.

**Table 1:** Characterization of hemodialysis patients

Sample characteristics	***n*** (%)	Mean (95% CI)
Gender *(%)*		
Male	49 (61.25)	
Female	31 (38.75)	
Age *(years)*		52.91 (49.36–56.47)
Duration of HD *(years)*		4.36 (3.45–5.27)
Diuresis Volume *(mL)*		435.91 (263.63–608.18)
Kt/v		1.46 (1.37–1.56)
Comorbidities		
Systemic hypertension	73 (91.30)	
Diabetes	20 (25.00)	
Obesity	11 (13.80)	
Others	27 (33.80)	
DXA		
Body fat *(%)*		29.21 (26.81–31.61)
Appendicular lean mass *(kg)*		18.04 (16.85–19.23)
BMD total *(g/cm*^*3*^*)*		0.99 (0.96–1.04)
BMD spine *(g/cm*^*3*^*)*		1.00 (0.95–1.07)
BMD hip *(g/cm*^*3*^*)*		0.83 (0.79–0.88)
Blood clinical biomarkers		
Vitamin D *(ng/mL)*		36.08 (32.70–39.46)
PTH *(pg/mL)*		343.09 (293.63–392.54)
CRP *(mL/L)*		9.29 (5.57–13.01)
Hb *(g/dL)*		10.83 (10.50–11.16)
Alkaline phosphatase *(U/L)*		134.30 (114.56–154.04)
Ferritin *(ng/mL)*		415.76 (343.57–487.95)

Data presented as mean and 95% CI or absolute number and percentage. *Abbreviations*: *n* number of patients, % percentage, *CI* confidence interval, *Kt/V* fractional urea clearance, *DXA* dual-energy x-ray absorptiometry, *BMD* bone mineral density, *PTH* parathormone, *CRP* C-reactive protein, *Hb* hemoglobin

**Table 2:** Physical functioning characterization of hemodialysis patients

Sample characteristics	Mean (95% CI)
Human Activity Profile *(points)*	44.37 (39.60–49.13)
*Body structure and function*	
HS *(kg)*	33.42 (31.02–35.83)
5-STS *(s)*	14.25 (12.84–15.66)
60-STS *(repetitions)*	18.96 (17.05–12. 87)
*Activity*	
SPPB *(points)*	8.91 (8.32–9.50)
*Social participation*	
Participation scale *(points)*	10.12 (7.86–12.39)

Data presented as mean and 95% CI or absolute number and percentage. *Abbreviations %* percentage, *CI* confidence interval, *HS* handgrip strength, *5-STS* the 5 repetition sit-to-stand test, *60-STS* the 60 s sit-to-stand test, *SPPB* Short Physical Performance Battery

Regarding the body structure and function domain, appendicular lean mass, PTH level and age were associated with HS in HD patients, explaining 55.8% of the variability in HS. Moreover, HAP and PTH were associated with 5-STS, explaining 26.3% of the variability in 5-STS. It was also observed that a reduction in HAP and PTH levels, and the increase in the duration of HD and an advanced age, were associated with low 60-STS performance, explaining 33.7% of the variability in 60-STS (Table 3). Regarding the activity domain, worse HAP, low PTH, high alkaline phosphatase, advancing of duration of HD and age, and body fat, explained 68.9% of the variability in SPPB (Table 3). Moreover, only HAP was associated with worse social participation, explaining 6.7% of the variability in the participation scale (Table 3).

**Table 3:** Multiple linear regression analysis of the hemodialysis patients

Model	Independent outcomes	Dependent outcomes	Beta	***p***-value	R^**2**^adjusted
*Body structure and function*					
	ALM (*Kg)*	HS *(Kg)*	0.69	0.000	0.558
	PTH (*pg/mL)*		0.23	0.009	
	Age (*years)*		−0.18	0.034	
	HAP (*points)*	5-STS *(s)*	−0.38	0.001	0.263
	PTH (*pg/mL)*		−0.32	0.007	
	HAP *(points)*	60-STS *(repetitions)*	0.39	0.001	0.337
	Duration of HD *(years)*		−0.24	0.025	
	PTH *(pg/mL)*		0.23	0.031	
	Age (*years*)		−0.23	0.039	
*Activity*					
	HAP (*points)*	SPPB *(points)*	0.43	0.000	0.689
	PTH (*pg/mL)*		0.34	0.000	
	Alkaline phosphatase *(U/L)*		−0.31	0.000	
	Duration of HD *(years)*		−0.29	0.001	
	Age (years*)*		−0.22	0.006	
	Body fat (*%)*		−0.19	0.030	
*Social participation*	HAP (*points)*	Participation scale *(points)*	−0.26	0.032	0.067

*ALM* Appendicular lean mass, *PTH* parathormone, *HAP* human activity profile, *HS* handgrip strength, *5-STS* five repetition sit-to-stand test, *60-STS* 60 s sit-to-stand test, *SPPB* Short Physical Performance Battery

## Discussion

ESRD is a complex disease, with systemic repercussions [2–6]. The uremic environment, inflammation, insulin resistance, metabolic acidosis, and alterations of mineral metabolism lead to PEW [3]. Furthermore, the disease results in loss of muscle mass, strength, and functionality [2, 3]. Additionally, the impact of the disease predisposes a more sedentary lifestyle [37] and increased risk of falls and frailty leading to further hospitalizations, poor quality of life, mortality, and morbidity [4].

This was the first study to describe factors associated with the functioning of HD patients in reference to the ICF. As expected, the findings revealed that lower HAP was a determinant of impaired body structure and function, and reduced activity and social participation in HD patients. These findings reinforce those of Jimenez et al., who showed an association between HAP and functional capacity [38]. However, the fact that we included body composition data, blood biomarkers, and confounding factors such as age, diuresis volume, and duration of HD in our analyses, although HAP was the main variable associated with all ICF domains, demonstrates that remaining physically active is crucial and probably more relevant than clinical aspects in the context of functionality [39, 40].

There is a consistent link between the reduction of physical activity and poor prognosis regardless of patient characteristics [41]. Thus, the challenge for health professionals is to tailor public policy and programs to promote increased physical activity for ESRD [42]. Furthermore, only HAP was a predictor of the ICF social participation domain. Social participation is the most complex domain of the ICF, being responsible for describing the person’s involvement in daily activities [7]. Some of the chapters used to describe social participation in the ICF are “Mobility, Self-care, and Domestic life” [26]. These chapters refer to elements present in the HAP questionnaire, so it explains the association between these variables. Despite the association being weak, it reinforces the importance of a physical activity routine for HD patients.

Although there is evidence that increased PTH levels could be associated with poor functioning [43], other studies pointed out the association between higher PTH levels and increased survival in HD patients [44, 45]. In this regard, our data demonstrated a positive association between higher PTH levels and better functioning in the ICF domains of body structure and function and activity in HD patients. A possible explanation could be attributed to the higher doses of vitamin D recommended in patients with high PTH levels in an attempt to contain secondary hyperparathyroidism [46], with vitamin D also having an important role in the regulation of the immune system, modulating both the inflammatory response [47] and musculoskeletal adaptations in HD patients [48]. Therefore, higher doses of vitamin D modulating inflammation and musculoskeletal adaptations may be behind the high PTH levels. However, the mean (95% CI) of vitamin D in our sample was [36.08 (32.70–39.46)], which is within the normal range. Thus, higher doses of vitamin D were not able to justify our findings.

In addition to the hypothesis raised, inflammation increases body energy expenditure at rest and suppresses anabolic hormones (like PTH), often being associated with PEW and muscle atrophy [2, 44, 45]. Thus, it is possible that the higher the PTH levels, the lower the inflammation, leading to better functioning in HD patients [49]. However, our data showed that the mean (95% CI) of CRP was [9.29 (5.57–13.01)], which is a high mean concentration of this inflammatory biomarker. Therefore, more studies are necessary to test these suppositions, and it is necessary to investigate other inflammatory biomarkers like IL-6 od and IL-1 beta, which have been shown to suppress PTH in laboratory studies [45]. Moreover, for perspective, future longitudinal studies should be designed to confirm if high PTH levels indicate lower inflammation in HD patients.

This study also showed that ALM was a predictor of upper limb strength (HS), which is included in the ICF body structure and function domain. According to previous studies, HS is independently and inversely associated with malnutrition and inflammation (which refers to PEW) in HD patients [50]. As mentioned above, PEW results in loss of muscle mass, strength, and functionality [2, 3], which might explain the association between HS and ALM.

Another important aspect from the present study is the association of high alkaline phosphatase levels with poor performance on the ICF activity domain. This is in line with other evidence that showed higher serum alkaline phosphatase levels associated with inactivity in subjects with and without kidney disease, which may be explained by the aforementioned mechanisms of inflammation [51]. It seems that higher serum alkaline phosphatase levels and inflammation are true protagonists of worse performance on physical functioning tests.

It is noteworthy that age and duration of HD, diuresis volume, and fractional urea clearance were used to adjust all multiple regression models. The current study showed that age and duration of HD were significantly associated with the ICF body structure and function and activity domains in HD patients. These results are in line with those of Johansen et al. [52], which showed age as a predictor of worse physical functioning, including gait speed, 5-STS, and physical activity level in HD patients. However, although Tsutsui et al. [53] showed that the duration of HD could affect HD patients’ self-reported physical functioning, this was the first study to show the association between duration of HD and body structure and function and activity domains in the context of the ICF.

The strength of this study is that it is the first to investigate factors associated with functioning within ICF domains in HD patients. In this sense, we concluded that regular physical activity is crucial for the maintenance of functioning in HD patients and probably more relevant than clinical aspects such as body composition or biomarker levels, demonstrating the importance of health professionals encouraging their patients to remain physically active. In addition, higher PTH levels were also a determinant of better functioning in the ICF domains of the HD patients; however, additional studies are needed to determine the explanation for this association. Finally, although ALM is directly associated with HS and body fat is inversely associated with SPPB, other body composition measurements were not associated with other ICF domains. Thus, body composition was not the protagonist of functioning in HD patients. Alkaline phosphatase, inflammation, and body composition may work as mediators of the associations found, but more studies are needed to prove this hypothesis.

This study had some limitations. Firstly, a longitudinal study is necessary to determinate the cause-effect of biomarkers, body composition, and physical activity level on functioning in the ICF context. Secondly, the results cannot be extrapolated to all kidney patients since this study was conducted only with HD patients. Thirdly, in future, studies using an accelerometer should be carried out to assess physical activity level in HD patients. Fourthly, because protein intake and depression are factors that might affect muscle strength, dietary intake and screening of depression should be evaluated in future studies. Finally, although we recognize that it is not recommended to assess physical function in HD patients after the weekend, handgrip strength was collected in the first hemodialysis session of the week, respecting the routine of tests already performed in the hospital’s hemodialysis sector on other days.

## Conclusion

Physical activity and PTH levels are determinant protagonists of functioning in all ICF domains in hemodialysis patients.

## Supplementary Information

**Additional file 1.** Raw data and calculated parameters.

**Additional file 2.** Correlations according to the sample distribution of hemodialysis patients.

**Additional file 3.** Simple linear regression analysis of hemodialysis patients.

## Data Availability

All data generated or analysed during this study are included in this published article [and its supplementary information files].
