# Supplementary material for: Determining factors of functioning in hemodialysis patients using the international classification of functioning, disability and health
Source: BMC Nephrol. 2022 Mar 24;23:119. doi: 10.1186/s12882-022-02719-5 (PMC8944099; doi:10.1186/s12882-022-02719-5)

Raw data.

| Identification | Obesity | Systemic hypertension | Diabetes | Others | Gender | Age (years) | Duration of HD (years) | Diuresis Volume (mL) | HS (kg) | 5-STS (s) |
| --- | --- | --- | --- | --- | --- | --- | --- | --- | --- | --- |
| V1 |  | 1 | 0 | 0 | 2 | 61 | 2.90 | 50 | 38 | 14.60 |
| V2 | 0 | 1 | 1 | 1 | 1 | 27 | 2.67 | 50 | 22 | 13.18 |
| V3 | 0 | 0 | 0 | 0 | 1 | 27 | 5.00 | 0 | 33 | 11.52 |
| V4 | 1 | 1 | 0 | 1 | 1 | 79 | 3.83 | 800 | 22 | 18.95 |
| V5 | 1 | 1 | 0 | 0 | 2 | 30 | 2.20 | 425 | 40 | 12.24 |
| V6 | 0 | 1 | 0 | 0 | 1 | 50 | 0.83 |  | 30 | 15.84 |
| V7 | 1 | 1 | 1 | 0 | 2 | 63 | 0.92 | 150 | 34 | 15.86 |
| V8 | 0 | 1 | 1 | 0 | 2 | 47 | 1.67 | 0 | 44 | 9.52 |
| V9 | 0 | 1 | 0 | 0 | 2 | 67 | 17.3 | 0 | 40 | 0.00 |
| V10 | 0 | 1 | 0 | 0 | 2 | 69 | 2.33 | 950 | 42 | 16.75 |
| V11 | 0 | 1 | 0 | 0 | 2 | 54 | 1.17 | 250 | 48 | 13.51 |
| V12 | 0 | 1 | 0 | 0 | 2 | 34 | 5.25 | 1550 | 36 | 14.22 |
| V13 | 0 | 1 | 0 | 0 | 2 | 47 | 6.50 | 100 | 38 |  |
| V14 | 0 | 1 | 0 | 1 | 2 | 59 | 1.25 |  | 44 | 11.65 |
| V15 | 0 | 1 | 0 | 1 | 2 | 46 | 2.75 | 200 | 37 | 13.45 |
| V16 | 1 | 1 | 0 | 0 | 1 | 61 | 1.58 | 350 | 30 | 10.3 |
| V17 | 0 | 1 | 1 | 1 | 1 | 78 | 5.75 | 0 | 21 | 0.00 |
| V18 | 0 | 1 | 0 | 1 | 1 | 20 | 0.83 |  | 24 | 12.64 |
| V19 | 0 | 0 | 1 | 1 | 2 | 66 | 9.67 | 50 | 32 | 11.99 |
| V20 | 0 | 1 | 0 | 0 | 1 | 41 | 1.58 |  | 34 | 16.20 |
| V21 | 0 | 1 | 0 | 0 | 1 | 26 | 3.17 |  | 30 | 10.48 |
| V22 | 0 | 1 | 0 | 1 | 2 | 30 | 1.25 |  | 40 | 12,00 |
| V23 | 0 | 1 | 1 | 0 | 2 | 47 | 0.25 | 2000 | 35 | 15.78 |
| V24 | 0 | 0 | 0 | 0 | 2 | 23 | 8.50 | 0 | 50 | 8.52 |
| V25 | 0 | 1 | 0 | 1 | 1 | 55 | 1.25 | 850 | 28 | 13,00 |
| V26 | 0 | 1 | 0 | 0 | 2 | 52 | 1.50 | 100 | 40 | 10.15 |
| V27 | 0 | 1 | 0 | 1 | 2 | 53 | 2.00 | 3250 | 41 | 13.49 |
| V28 | 0 | 0 | 0 | 0 | 2 | 45 | 8.50 | 0 | 68 | 9.49 |
| V29 | 0 | 1 | 0 | 0 | 1 | 39 | 9.70 | 0 | 20 | 28.66 |
| V30 | 0 | 1 | 0 | 0 | 2 | 39 | 6.08 | 0 | 17 | 15.24 |
| V31 | 0 | 1 | 0 | 1 | 1 | 76 | 4.58 | 100 | 23 | 12.59 |
| V32 | 0 | 1 | 0 | 1 | 2 | 78 | 0.42 | 300 | 40 | 24.80 |
| V33 | 0 | 1 | 0 | 0 | 2 | 59 | 1.17 |  | 34 | 20.22 |
| V34 | 0 | 1 | 0 | 0 | 2 | 71 | 2.75 | 1650 | 31 | 10.44 |
| V35 | 0 | 1 | 0 | 0 | 2 | 71 | 6.25 | 0 | 36 | 13.58 |
| V36 | 0 | 1 | 0 | 0 | 2 | 52 | 2.17 | 20 | 41 | 13.21 |
| V37 | 0 | 1 | 1 | 0 | 2 | 59 | 9.75 | 0 | 32 | 14.45 |
| V38 | 0 | 1 | 0 | 0 | 2 | 49 | 5.17 |  | 38 | 10.66 |
| V39 | 0 | 1 | 0 | 0 | 2 | 52 | 0.58 | 0 | 41 | 14.34 |
| V40 | 0 | 1 | 0 | 1 | 2 | 65 | 4.33 | 700 | 40 |  |
| V41 |  | 1 | 0 | 0 | 2 | 79 | 8.58 | 0 | 32 | 33.73 |
| V42 | 1 | 1 | 0 | 0 | 2 | 59 | 1.83 |  | 50 | 14.99 |
| V43 | 0 | 0 | 1 | 0 | 2 | 57 | 15.5 | 0 | 30 | 24,00 |
| V44 | 1 | 1 | 0 | 1 | 2 | 78 | 1.75 | 2100 | 30 | 16.09 |
| V45 | 0 | 1 | 1 | 0 | 2 | 48 | 0.92 | 0 | 30 | 14.69 |
| V46 | 0 | 1 | 1 | 1 | 2 | 67 | 2.08 | 200 | 36 | 13.02 |
| V47 | 0 | 1 | 0 | 0 | 2 | 27 | 1.83 | 300 | 58 | 11.01 |
| V48 | 0 | 1 | 0 | 0 | 2 | 29 | 7.17 | 0 | 50 | 12.14 |
| V49 | 0 | 1 | 1 | 1 | 1 | 63 | 1.80 | 1250 | 23 | 14.96 |
| V50 | 0 | 1 | 1 | 1 | 2 | 57 |  | 0 | 48 | 14.59 |
| V51 | 0 | 1 | 0 | 0 | 2 | 54 | 1.50 | 2200 | 52 | 15.43 |
| V52 | 0 | 1 | 1 | 0 | 1 | 66 | 4.08 | 50 | 30 | 16.72 |
| V53 | 1 | 1 | 1 | 0 | 1 | 66 | 1.92 | 450 | 22 | 0.00 |
| V54 | 1 | 1 | 0 | 1 | 1 | 56 | 4.17 | 0 | 21 | 22.39 |
| V55 | 0 | 0 | 0 | 0 | 1 | 63 | 1.50 | 2400 | 20 |  |
| V56 |  | 1 | 0 | 1 | 1 | 90 | 6.92 | 275 | 24 | 0.00 |
| V57 | 0 | 1 | 1 | 1 | 1 | 55 | 1.75 |  | 22 | 17.38 |
| V58 | 0 | 1 | 0 | 0 | 1 | 45 | 2.42 | 200 | 28 | 15.98 |
| V59 | 0 | 1 | 1 | 0 | 1 | 50 | 2.42 | 800 | 20 | 19.79 |
| V60 | 0 | 1 | 0 | 0 | 1 | 71 | 5.16 | 0 | 24 | 12.59 |
| V61 | 0 | 1 | 0 | 0 | 1 | 55 | 14.30 | 0 | 8 | 30.20 |
| V62 | 0 | 1 | 0 | 1 | 2 | 62 | 4.25 | 450 | 40 | 9.45 |
| V63 | 0 | 1 | 0 | 0 | 2 | 52 | 10.40 |  | 42 | 13.10 |
| V64 | 0 | 1 | 0 | 0 | 2 | 79 | 2.08 | 950 | 38 | 20.17 |
| V65 | 0 | 1 | 1 | 1 | 1 | 70 | 19.25 | 200 | 17 | 19.02 |
| V66 | 1 | 1 | 0 | 0 | 1 | 65 | 5.58 |  | 24 | 14.33 |
| V67 | 1 | 1 | 0 | 0 | 1 | 63 | 5.42 | 1400 | 21 | 10.80 |
| V68 | 1 | 1 | 1 | 0 | 1 | 39 | 2.00 | 100 | 34 | 14.68 |
| V69 | 0 | 1 | 0 | 0 | 2 | 53 | 8.92 | 0 | 50 |  |
| V70 | 0 | 1 | 0 | 1 | 2 | 48 | 1.50 | 500 | 42 | 18.04 |
| V71 | 0 | 1 | 0 | 1 | 2 | 39 | 2.00 | 0 | 40 | 12.71 |
| V72 |  | 1 | 0 | 0 | 1 | 29 | 2.08 | 0 | 22 | 16.77 |
| V73 | 0 | 1 | 0 | 0 | 1 | 34 | 1.75 |  | 18 | 0.00 |
| V74 | 0 | 1 | 0 | 1 | 2 | 35 | 2.17 | 550 | 38 | 12.80 |
| V75 | 0 | 1 | 0 | 0 | 1 | 56 | 1.40 | 50 | 19 | 14.37 |
| V76 | 0 | 1 | 0 | 1 | 1 | 29 | 14.08 | 0 | 22 | 11.58 |
| V77 | 0 | 1 | 1 | 0 | 2 | 52 | 9.00 | 0 | 38 | 15.79 |
| V78 |  | 0 | 0 | 0 | 2 | 26 | 3.83 | 100 | 39 | 14.10 |
| V79 | 0 | 1 | 1 | 0 | 2 | 40 | 4.08 |  | 40 | 12.34 |
| V80 | 0 | 1 | 0 | 1 | 1 | 60 | 1.75 | 350 | 18 | 30,00 |

| Identification | 60-STS (repetitions) | SPPB | Participation scale (points) | Weight (kg) | Height (m) | BMI | BMD total (g/cm^3^) | BMD spine (g/cm^3^) | BMD hip (g/cm^3^) | Body fat (%) |
| --- | --- | --- | --- | --- | --- | --- | --- | --- | --- | --- |
| V1 | 23 | 10 | 4 | 55.20 | 1.66 |  |  |  |  |  |
| V2 | 21 | 8 | 1 | 44.80 | 1.53 | 19.10 | 0.92 | 0.85 | 0.73 | 38.1 |
| V3 | 33 | 11 | 10 | 46.10 | 1.54 | 19.60 | 0.88 | 0.85 | 0.70 | 26.4 |
| V4 | 15 | 5 | 40 | 69.80 | 1.54 | 29.80 | 0.89 | 0.94 | 0.66 | 42.6 |
| V5 | 25 | 11 | 10 | 100.10 | 1.83 | 33.20 | 1.11 | 1.06 | 0.88 | 33.2 |
| V6 | 23 | 10 | 4 | 68.20 | 1.57 | 27.70 | 0.94 | 0.91 | 0.84 | 40.1 |
| V7 | 17 | 7 | 0 | 85.60 | 1.61 | 33.00 | 1.1 | 1.38 | 0.88 | 44.4 |
| V8 | 31 | 12 | 8 | 71.50 | 1.73 | 23.80 | 1.05 | 0.89 | 0.82 | 18.4 |
| V9 | 0 | 4 | 27 | 45.60 | 1.47 | 21.10 | 0.76 | 1.16 | 0.59 | 24.0 |
| V10 | 8 | 5 | 9 | 95.30 | 1.67 | 24.40 |  |  |  |  |
| V11 | 24 | 11 | 11 | 76.20 | 1.64 | 28.30 | 1.39 | 1.51 | 1.21 | 28.1 |
| V12 | 21 | 9 | 4 | 48.10 | 1.60 | 19.00 | 0.93 | 0.97 | 0.82 | 22.5 |
| V13 | 22 | 11 | 0 | 55.00 | 1.69 | 19.50 | 0.93 | 0.91 | 0.78 | 12.9 |
| V14 | 24 | 11 | 1 | 94.70 | 1.80 | 29.10 | 1.36 | 1.52 | 1.41 | 33.5 |
| V15 | 23 | 11 | 5 | 65.80 | 1.79 | 20.50 | 1.17 | 1.15 | 0.98 | 17.5 |
| V16 | 28 | 12 | 1 | 85.20 | 1.50 | 37.90 | 1.01 | 1.12 | 0.91 | 37.9 |
| V17 | 0 | 3 | 21 | 41.00 | 1.44 | 20.00 | 0.72 | 0.64 | 0.49 | 27.8 |
| V18 | 22 | 11 | 29 | 49.90 | 1.57 | 20.20 | 1.06 | 0.97 | 0.86 | 32.0 |
| V19 | 28 | 11 | 0 | 56.30 | 1.57 | 22.80 | 0.79 | 0.83 | 0.64 | 27.6 |
| V20 | 18 | 10 | 14 | 58.70 | 1.55 | 24.40 | 1.02 | 0.9 | 0.82 | 45.4 |
| V21 | 25 | 12 | 10 | 57.20 | 1.58 | 22.90 | 1.03 | 0.93 | 0.89 | 38.1 |
| V22 | 26 | 11 | 13 | 59.00 | 1.60 | 23,00 | 1.15 | 1.14 | 1.04 | 15.2 |
| V23 | 18 | 10 | 12 | 68.60 | 1.70 | 24.20 | 1.06 | 1.02 | 0.84 | 23.1 |
| V24 | 38 | 11 | 0 | 56.60 | 1.68 | 20.10 | 1.12 | 0,00 | 0.95 | 19.9 |
| V25 | 22 | 11 | 5 | 42.90 | 1.48 | 19.60 | 0.85 | 0.97 | 0.73 | 30.7 |
| V26 | 30 | 12 | 23 | 64.90 | 1.68 | 23,00 | 1.08 | 1.03 | 0.93 | 28.8 |
| V27 | 21 | 10 | 12 | 67.20 | 1.66 | 24.40 | 0.96 | 0.99 | 0.85 | 25.4 |
| V28 | 29 | 12 | 15 | 76.50 | 1.66 | 27.80 | 1.16 | 1.34 | 1.08 | 31.2 |
| V29 | 0 | 2 | 18 | 48.90 | 1.52 | 21.20 | 0.88 | 0.89 | 0.72 | 29.7 |
| V30 | 16 | 8 | 0 | 48.60 | 1.57 | 19.70 | 0.76 | 0.83 | 0.63 | 14.6 |
| V31 | 24 | 6 | 21 | 42.00 | 1.52 | 18.20 | 0.74 | 0.71 | 0.47 | 28.2 |
| V32 | 6 | 7 | 5 | 79.70 | 1.72 | 27.30 | 0.8 | 0.92 | 0.58 | 15.7 |
| V33 | 15 | 9 | 12 | 61.20 | 1.60 | 24,00 | 0.97 | 1.09 | 0.79 | 32.8 |
| V34 | 28 | 12 | 1 | 61.10 | 1.66 | 22.40 | 0.95 | 0.87 | 0.81 | 18.9 |
| V35 |  | 8 | 8 | 63.50 | 1.72 | 21.70 | 1.17 | 1.4 | 0.98 | 28.8 |
| V36 | 22 | 11 | 25 | 69.80 | 1.65 | 25.60 | 1.04 | 1.09 | 0.82 | 37.6 |
| V37 | 20 | 10 | 5 | 58.60 | 1.67 | 21,00 | 0.75 | 0.77 | 0.65 | 17.7 |
| V38 | 23 | 12 | 10 | 54.70 | 1.66 | 19.80 | 0.99 | 1.03 | 0.79 | 20.3 |
| V39 | 21 | 10 | 4 | 66.80 | 1.71 | 22.80 | 1.2 | 1.07 | 1.06 | 26.4 |
| V40 |  | 8 | 1 | 75.80 | 1.70 | 26.20 | 1.19 | 1.22 | 0.96 | 37.1 |
| V41 | 6 | 7 | 1 | 61.50 | 1.64 |  |  |  |  |  |
| V42 | 20 | 10 | 8 | 79.30 | 1.63 | 29.80 | 1185,00 | 0.988 | 0.90 | 33.1 |
| V43 | 6 | 9 | 12 | 53.20 | 1.76 | 17.20 | 1002,00 | 1485,00 | 1.06 | 10.9 |
| V44 | 16 | 7 | 0 | 74.60 | 1.58 | 29.70 | 1.02 | 1.23 | 0.80 | 45.1 |
| V45 | 18 | 10 | 8 | 79.90 | 1.77 | 25.50 | 1302,00 | 1162,00 | 1.06 | 20.9 |
| V46 | 22 | 11 | 1 | 58.80 | 1.62 | 22.70 | 1021,00 | 1064,00 | 0.79 | 19.7 |
| V47 | 29 | 12 | 0 | 64.70 | 1.72 | 21.90 | 1165,00 | 1128,00 | 0.96 | 11.7 |
| V48 | 22 | 11 | 18 | 56.50 | 1.68 | 20.00 | 1002,00 | 0.994 | 0.82 | 12.8 |
| V49 |  | 9 | 23 | 50.50 | 1.44 | 24.70 | 0.914 | 0.89 | 0.79 | 27.1 |
| V50 | 21 | 10 | 0 | 60.20 | 1.59 | 23.80 | 0.859 | 0.825 | 0.66 | 18.9 |
| V51 | 20 | 10 | 2 | 81.50 | 1.70 | 28.20 | 1265,00 | 1372,00 | 1.23 | 30.7 |
| V52 | 18 | 5 | 33 | 53.10 | 1.53 | 22.10 | 0.65 | 0.719 | 0.45 | 37.5 |
| V53 | 0 | 4 | 8 | 80.30 | 1.56 | 33,00 | 1034,00 | 1035,00 | 0.95 | 47.9 |
| V54 | 13 | 5 | 8 | 79.20 | 1.55 | 33.40 | 1267,00 | 1417,00 | 1.19 | 44.3 |
| V55 |  |  | 13 | 65.20 | 1.59 | 25.80 | 1021,00 | 0.957 | 0.86 | 38.2 |
| V56 | 0 | 4 | 3 | 62.60 | 1.58 |  |  |  |  |  |
| V57 | 19 | 7 | 18 | 57.90 | 1.45 | 27.90 | 0.86 | 0.9 | 0.74 | 43.0 |
| V58 | 18 | 10 | 20 | 51.10 | 1.56 | 21.30 | 0.906 | 0.911 | 0.77 | 35.2 |
| V59 | 14 | 3 | 0 | 56.70 | 1.55 | 23.60 | 0.891 | 0.933 | 0.65 | 36.4 |
| V60 | 24 | 10 | 1 | 52.30 | 1.52 | 22.60 | 0.676 | 0.766 | 0.51 | 31.4 |
| V61 | 8 | 8 | 3 | 41.90 | 1.52 | 18.10 | 0.786 | 0.836 | 0.70 | 25.0 |
| V62 | 30 | 11 | 17 | 69.00 | 1.61 | 26.60 | 1146,00 | 1291,00 | 0.95 | 25.7 |
| V63 | 22 | 11 | 10 | 63.00 | 1.70 | 27.50 | 0.975 | 0.972 | 0.76 | 17.3 |
| V64 | 10 | 9 | 8 | 57.00 | 1.60 | 22.10 | 1105,00 | 1157,00 | 0.78 | 36.6 |
| V65 | 14 | 4 | 15 | 40.30 | 1.53 | 21.10 | 0.928 | 0.801 | 0.71 | 21.1 |
| V66 | 21 | 6 | 0 | 67.60 | 1.41 | 34.00 | 0.969 | 1015,00 | 0.79 | 43.8 |
| V67 | 23 | 8 | 0 | 82.00 | 1.47 | 37.90 | 0.926 | 0.994 | 0.85 | 51.6 |
| V68 | 19 | 10 | 21 | 73.60 | 1.54 | 31.30 | 1301,00 | 1385,00 | 1.14 | 43.4 |
| V69 |  |  | 0 | 87.20 | 1.75 | 28.50 | 1147,00 | 1288,00 | 1.07 | 29.1 |
| V70 | 16 | 9 | 17 | 68.20 | 1.54 | 29.10 | 1116,00 | 1297,00 | 0.92 | 33.9 |
| V71 | 22 | 11 | 30 | 66.00 | 1.77 | 21.30 | 1016,00 | 0.959 | 0.82 | 24.0 |
| V72 | 16 | 8 | 7 | 50.90 | 1.53 |  |  |  |  |  |
| V73 | 0 | 4 | 53 | 52.00 | 1.64 | 19.80 | 0.948 | 0.944 | 0.71 | 22.9 |
| V74 | 24 | 11 | 14 | 50.20 | 1.66 | 18.70 | 0.913 | 0.00073 | 0.63 | 17.6 |
| V75 | 24 | 8 | 5 | 56.50 | 1.42 | 28.00 | 0.88 | 0.958 | 0.80 | 52.5 |
| V76 | 24 | 11 | 4 | 39.10 | 1.43 | 19.10 | 1066,00 | 1129,00 | 1.07 | 15.6 |
| V77 | 19 | 10 | 6 | 70.70 | 1.64 | 26.60 | 1053,00 | 1078,00 | 0.92 | 33.1 |
| V78 | 18 | 10 | 8 |  |  |  |  |  |  |  |
| V79 | 24 | 11 | 10 | 44.50 | 1.54 | 18.80 | 0.937 | 0.94 | 0.73 | 13.1 |
| V80 | 12 | 6 | 6 | 55.30 | 1.40 | 28.20 | 0.768 | 0.861 | 0.60 | 37.9 |

| Identification | Appendicular lean mass (kg) | Vitamin D (ng/mL) | PTH (pg/mL) | CRP (mL/L) | HAP(points) | ktv | Hb (g/dL) | Ferritin (ng/mL) | Alkaline phosphatase (U/L) |
| --- | --- | --- | --- | --- | --- | --- | --- | --- | --- |
| V1 |  | 26,50 | 256,20 | 5,94 | 27 | 1.41 | 9.20 | 234 | 62 |
| V2 | 10.57 | 51,70 | 231,50 | 1,87 | 18 | 1.78 | 10.40 | 215 | 103 |
| V3 | 12.55 | 29,40 | 308,60 | 2,63 | 61 | 1.63 | 9.80 | 497 | 71 |
| V4 | 17.32 | 15,90 | 161,80 | 3,45 | 10 | 1.37 | 12.30 | 316 | 82 |
| V5 | 33.92 | 34,60 | 187,40 | 2,77 | 67 |  | 9.60 | 411 | 73 |
| V6 | 17.21 | 33,20 | 339,80 | 30,75 | 37 | 1.6 | 11.30 | 1749 | 67 |
| V7 | 19.27 | 41,30 | 547,20 | 2,47 |  | 1.2 | 9.90 |  | 69 |
| V8 | 25.87 | 56,60 | 314,70 | 114,77 | 45 | 1.21 | 11.70 | 202 | 41 |
| V9 | 13.52 | 43,20 | 478,50 | 15,73 | 15 | 1.72 | 13.50 | 1067 | 208 |
| V10 |  | 46,30 | 201,10 | 3,35 | 58 | 1.11 | 10.30 | 475 | 99 |
| V11 | 23.87 | 42,50 | 311,50 | 3,54 | 65 | 1.23 | 10.10 | 125 | 53 |
| V12 | 13.99 | 38,80 | 189,80 | 15,81 | 40 | 1.69 | 10.80 | 100 | 110 |
| V13 | 19.23 | 25,70 | 806,90 | 3,90 | 75 | 1.4 | 9.40 | 287 | 247 |
| V14 | 28.92 | 42,10 | 135,10 | 5,65 | 40 | 1.17 | 12.10 | 152 | 180 |
| V15 | 22.84 | 28,30 | 229,30 | 71,57 | 61 | 1.15 | 9.80 | 331 | 48 |
| V16 | 19.52 | 53,90 | 823,60 | 14,90 | 46 | 1.33 | 9.30 | 290 | 75 |
| V17 | 11.71 | 49,20 | 120,30 | 2,44 | 48 | 1.6 | 9.80 | 269 | 101 |
| V18 | 11.91 | 33,60 | 204,40 | 3,54 | 26 | 2.94 | 9.00 | 210 | 92 |
| V19 | 15.96 | 73,40 | 509,30 | 15,55 | 49 | 1.56 | 11.30 | 497 | 119 |
| V20 | 12.89 | 34,20 | 383,70 | 2,15 | 54 | 1.69 | 12,00 | 683 | 121 |
| V21 | 14.43 | 30,40 | 393,70 | 3,88 | 70 | 3.03 | 9.50 | 94 | 200 |
| V22 | 20.01 | 37,40 | 93,00 | 3,38 | 72 | 1.06 | 9.70 | 315 | 101 |
| V23 | 24.49 | 38,40 | 225,80 | 18,24 | 38 | 0.71 | 10.30 | 108 | 51 |
| V24 | 19.05 | 35,40 | 510,40 | 2,65 | 72 | 1.46 | 11.70 | 714 | 299 |
| V25 | 10.75 | 46,50 | 998,60 | 24,19 | 25 | 1.21 | 10.40 | 65 | 229 |
| V26 | 18.76 | 34,30 | 236,80 | 3,70 | 67 | 1.17 | 11.40 | 61 | 92 |
| V27 | 20.98 | 40,00 | 219,60 | 9,38 | 30 | 1.16 | 11.10 | 32 | 150 |
| V28 | 24.68 | 37,60 | 740,60 | 4,36 | 53 | 1.34 | 11.60 | 561 | 83 |
| V29 | 13.53 | 31,80 | 158,50 | 5,08 | 42 | 1.5 | 10.80 | 393 | 504 |
| V30 | 16.04 | 39,00 | 433,60 | 10,99 | 75 | 1.63 | 12.40 | 231 | 365 |
| V31 | 12.26 | 20,00 | 263,90 | 5,84 | 42 | 1.67 | 11.50 | 751 | 83 |
| V32 | 26.46 | 23,20 | 381,70 | 3,90 | 2 | 1.13 | 11.90 | 263 | 154 |
| V33 | 17.63 | 40,90 | 224,10 | 5,15 | 49 | 1.38 | 10.60 | 117 | 161 |
| V34 | 19.84 | 44,20 | 320,90 | 2,56 | 68 | 1.3 | 12.30 | 51 | 72 |
| V35 | 19.24 | 32,50 | 390,50 | 1,62 | 25 | 1.33 | 11.20 | 474 | 80 |
| V36 | 18.49 | 32,30 | 285,30 | 5,96 | 38 | 1.46 | 13.70 | 574 | 85 |
| V37 | 19.44 | 28,90 | 352,10 | 4,32 | 70 | 1.24 | 12.80 | 317 | 257 |
| V38 | 17.28 | 38,10 | 702,80 | 2,64 | 60 | 1.52 | 7.60 | 1183 | 81 |
| V39 | 20.95 | 3,40 | 0,00 | 3,60 | 62 | 1.21 | 14.40 | 71 | 61 |
| V40 | 20.19 | 55,20 | 268,70 | 11,93 | 44 | 1.35 | 13.00 | 502 | 109 |
| V41 |  | 50,10 | 494,40 | 1,57 | 47 | 1.58 | 11.50 | 512 | 177 |
| V42 | 24.29 | 40,40 | 550,40 | 3,75 | 64 | 1.18 | 11.20 | 168 | 63 |
| V43 | 31.19 | 43,20 | 48,50 | 3,03 | 71 | 1.62 | 9.20 | 1422 | 67 |
| V44 | 16.46 | 41,60 | 261,40 | 2,19 | 54 | 1.36 | 11.80 | 422 | 88 |
| V45 | 21.84 | 44,60 | 16,50 | 3,22 | 30 | 1.11 | 7.80 | 415 | 170 |
| V46 | 18.16 | 22,30 | 185,80 | 5,89 | 45 | 1.22 | 11.40 | 71 | 81 |
| V47 | 24.22 | 53,70 | 323,80 | 1,83 | 80 | 1.29 | 9.90 | 475 | 93 |
| V48 | 20.08 | 69,40 | 484,60 | 12,15 | 12 | 1.28 | 9.20 | 525 | 182 |
| V49 | 13.26 | 14,30 | 68,30 | 2,53 | 12 | 1.74 | 6.80 | 581 | 68 |
| V50 | 20.88 | 54,30 | 494,70 | 5,02 | 63 | 1.35 | 12.10 | 714 | 130 |
| V51 | 25.79 | 42,10 | 299,70 | 5,39 | 49 | 1,00 | 11.10 | 236 | 44 |
| V52 | 12.83 | 20,00 | 336,60 | 1,75 | 7 | 1.81 | 11.50 | 1068 | 395 |
| V53 | 17.54 | 21,00 | 269,60 | 5,70 | 21 | 1.54 | 9.70 | 300 | 119 |
| V54 | 17.21 | 14,50 | 342,90 | 43,64 | 0 | 1.51 | 10.90 | 524 | 135 |
| V55 | 16.84 | 26,50 | 227,00 | 3,75 | 49 | 1.24 | 10.50 | 63 | 84 |
| V56 |  | 28,30 | 150,60 | 3,88 | 19 | 1.73 | 9.70 | 537 | 85 |
| V57 | 12.45 | 47,80 | 43,90 | 40,70 | 19 | 1.75 | 7.20 | 1314 | 125 |
| V58 | 14.27 | 27,70 | 628,40 | 4,82 | 34 | 0.98 | 10.50 | 166 | 100 |
| V59 | 12.69 | 21,50 | 108,20 | 2,49 |  | 0.85 | 9.20 | 410 | 102 |
| V60 | 14.19 | 32,10 | 573,60 | 1,81 | 60 | 1.44 | 12.00 | 140 | 279 |
| V61 | 11.7 | 17,50 | 130,30 | 18,32 | 52 | 1.54 | 10.00 | 790 | 119 |
| V62 | 20.89 |  |  |  | 59 | 3.31 | 10.40 | 279 | 160 |
| V63 | 21.72 | 39,70 | 388,20 | 3,84 | 54 | 1.4 | 12.00 | 512 | 93 |
| V64 | 14.54 | 29,70 | 257,60 | 23,61 | 52 | 1.41 | 9.80 | 307 | 92 |
| V65 | 11.06 | 34,80 | 221,90 | 1,93 | 12 | 1.83 | 11.10 | 214 | 91 |
| V66 | 15.19 | 16,50 | 339,60 | 5,69 | 10 | 1.41 | 11.00 | 801 | 82 |
| V67 | 14.49 | 16,00 | 654,70 | 3,10 |  | 1.58 | 12.70 | 425 | 337 |
| V68 | 17.38 | 26,40 | 1030,60 | 2,25 | 39 | 1.19 | 11.90 | 313 | 229 |
| V69 | 27.83 | 35,00 | 302,50 | 5,90 |  | 1.07 | 12.50 | 238 | 73 |
| V70 | 19.4 | 19,40 | 134,60 | 2,22 | 51 | 1.01 | 9.80 | 284 | 188 |
| V71 | 20.73 | 33,10 | 717,10 | 4,47 | 60 | 1.29 | 11.70 | 553 | 162 |
| V72 |  | 30,70 | 209,40 | 0,84 | 45 | 1.45 | 13.10 | 209 | 55 |
| V73 | 13.95 | 30,40 | 180,40 | 2,41 | 1 | 1.41 | 9.00 | 419 | 110 |
| V74 | 17.32 |  |  |  | 57 | 1.59 | 10.40 | 266 | 72 |
| V75 | 9.39 | 24,30 | 162,10 | 4,31 | 58 | 2.25 | 10,00 | 306 | 291 |
| V76 | 12.06 | 38,00 | 628,40 | 10,22 | 60 | 1.65 | 10.90 | 361 | 111 |
| V77 | 19.16 | 42,20 | 534,40 | 5,87 | 37 | 1.35 | 13.50 | 375 | 152 |
| V78 |  |  |  |  | 69 | 1.47 | 13.20 | 237 | 291 |
| V79 | 15.69 | 106,00 | 422,20 | 1,18 | 60 | 1.6 | 11.80 | 475 | 54 |
| V80 | 12.61 | 33,00 | 253,40 | 7,80 | 14 | 1.67 | 8.90 | 431 | 157 |

Legend: HS: handgrip strength; 5-STS: the 5 repetition sit-to-stand test; 60-STS: the 60 seconds sit-to-stand test; SPPB: Short Physical Performance Battery; BMI: body mass index; BMD: bone mineral density; PTH: parathormone; CRP: C-reactive protein; HAP: human activity profile; Kt/V: fractional urea clearance; Hb: hemoglobin.

**The Handgrip Strength analyses**


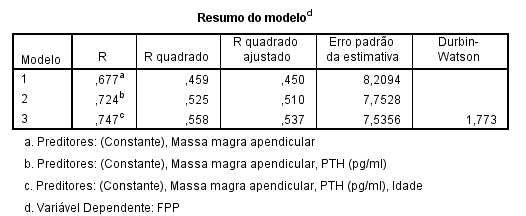


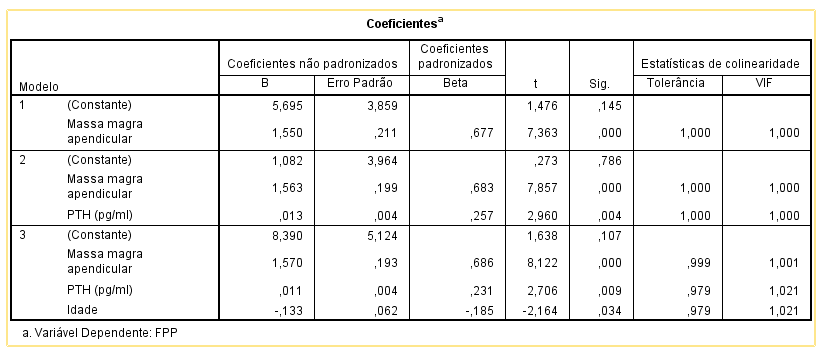


**The 5 repetition sit-to-stand test analyses**


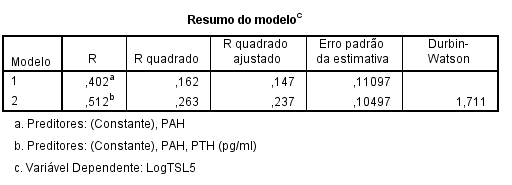


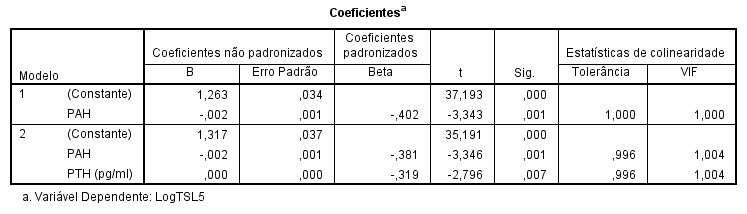


**The 60 seconds sit-to-stand test analyses**


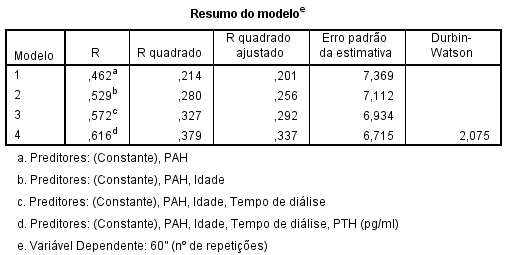


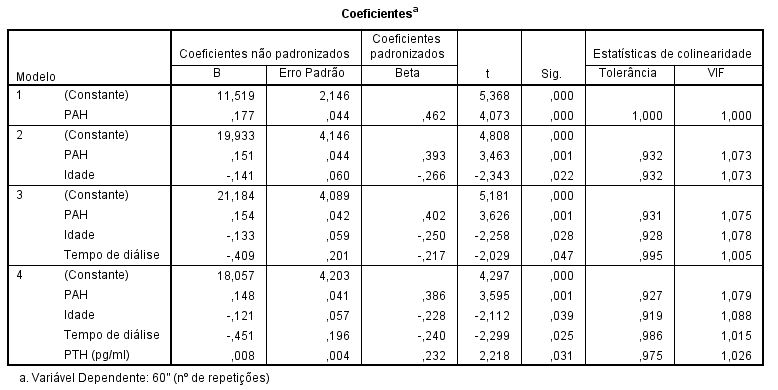


**The Short Physical Performance Battery analyses**


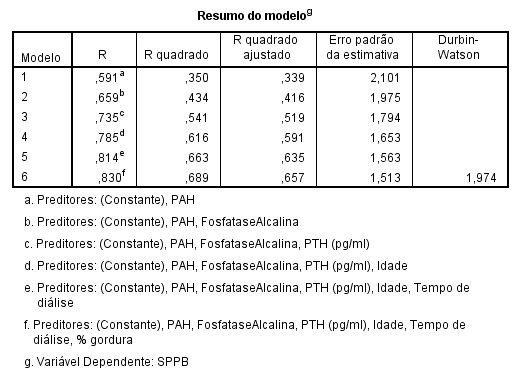

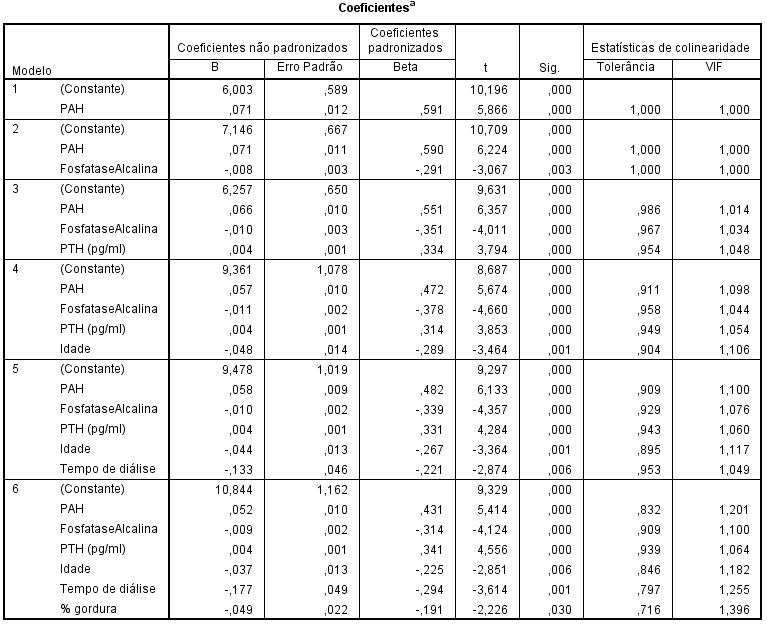


**The Participation Scale Analyses**


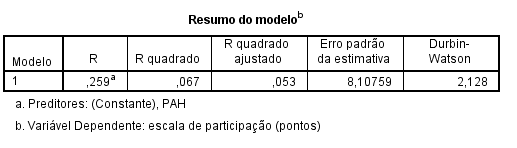


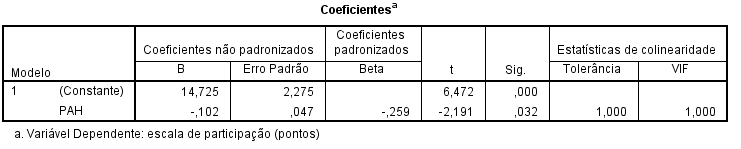

Supplement: Supplementary file 1 — Additional file 1. Raw data and calculated parameters. [file 12882_2022_2719_MOESM1_ESM.docx]
