# Supplementary material for: Determining factors of functioning in hemodialysis patients using the international classification of functioning, disability and health
Source: BMC Nephrol. 2022 Mar 24;23:119. doi: 10.1186/s12882-022-02719-5 (PMC8944099; doi:10.1186/s12882-022-02719-5)
Supplement: Supplementary file 2 — Additional file 2. Correlations according to the sample distribution of hemodialysis patients. [file 12882_2022_2719_MOESM2_ESM.docx]

Correlations according to the sample distribution of hemodialysis patients

| Chronic Kidney Disease Patients on Hemodialysis *(mean/ DP)* | HS, Kg | | 5-STS, s | | 60-STS,  repetitions | | SPPB, points | | Participation scale, points | |
| --- | --- | --- | --- | --- | --- | --- | --- | --- | --- | --- |
|  | r | p | r | p | r | p | r | p | r | p |
|  |  |  |  |  |  |  |  |  |  |  |
| Age (*years)* | -0.184 | 0.102 | **0.348** | **0.003** | **-0.403** | **0.000** | **-0.437** | **0.000** | -0.096 | 0.399 |
| Duration of HD (years*)* | -0.122 | 0.284 | 0.180 | 0.136 | -0.227 | 0.052 | -0.220 | 0.054 | -0.042 | 0.711 |
| Diuresis Volume*(mL)* | 0.000 | 1.000 | -0.034 | 0.798 | 0.019 | 0.884 | 0.023 | 0.855 | -0.056 | 0.655 |
| BMI | 0.058 | 0.623 | 0.017 | 0.889 | 0.024 | 0.844 | -0.090 | 0.449 | -0.175 | 0.134 |
| Body fat (*%)* | **-0.328** | **0.004** | 0.181 | 0.146 | -0.130 | 0.288 | **-0,345** | **0.003** | 0.033 | 0.779 |
| Appendicular lean mass (*Kg)* | **0.675** | **0.000** | -0.173 | 0.164 | 0.200 | 0.100 | **0.424** | **0.000** | -0.193 | 0.099 |
| BMD total (g/cm^3^) | **0.509** | **0.000** | **-0.259** | **0.036** | **0.273** | **0.023** | **0.382** | **0.001** | -0.133 | 0.258 |
| BMD spine (g/cm^3^) | **0.236** | **0.043** | 0.097 | 0.438 | -0.125 | 0.305 | 0.072 | 0.550 | -0.064 | 0.589 |
| BMD hip (g/cm^3^) | **0.426** | **0.000** | -0.230 | 0.064 | **0.256** | **0.034** | 0.392 | 0.001 | -0.216 | 0.064 |
| D Vitamin (*ng/mL)* | **0.332** | **0.003** | -0.237 | 0.052 | 0.181 | 0.129 | 0.280 | 0.015 | -0.154 | 0.181 |
| PTH (*pg/mL)* | 0,223 | 0.051 | **-0.314** | **0.009** | **0.283** | **0.016** | **0.341** | **0.003** | -0.117 | 0.310 |
| CRP *(mL/L)* | 0.024 | 0.837 | -0.070 | 0.573 | 0.132 | 0.270 | 0.122 | 0.299 | -0.079 | 0.496 |
| Kt/V | **-0.284** | **0.011** | -0.132 | 0.275 | 0.063 | 0.592 | -0.019 | 0.867 | 0.180 | 0.112 |
| Hb (*g/dL)* | **0.227** | **0.043** | -0.082 | 0.494 | 0.088 | 0.455 | 0.041 | 0.719 | -0.120 | 0.290 |
| Alkaline phosphatase *(U/L)* | -0.197 | 0.080 | 0.135 | 0.260 | -0.126 | 0.281 | -0.215 | 0.059 | 0.029 | 0.801 |
| Ferritin (*ng/mL)* | -0.061 | 0.591 | 0.174 | 0.150 | -0.136 | 0.248 | -0.184 | 0.110 | 0.127 | 0.264 |
| HAP | **0.370** | **0.001** | **-0.366** | **0.002** | **0.443** | **0.000** | **0.569** | **0.000** | **-0.373** | **0.001** |
|  |  |  |  |  |  |  |  |  |  |  |
| BMI: body mass index; BMD: bone mineral density; PTH: parathormone; CRP: C-reactive protein; Kt/V: fractional urea clearance; Hb: hemoglobin; HAP: human activity profile; HS: handgrip strength; 5STS: 5 repetitions sit-to-stand test; 60STS: 60 seconds sit-to-stand test; SPPB: Short Physical Performance Battery. | | | | | | | | | | |
