# Supplementary material for: Determining factors of functioning in hemodialysis patients using the international classification of functioning, disability and health
Source: BMC Nephrol. 2022 Mar 24;23:119. doi: 10.1186/s12882-022-02719-5 (PMC8944099; doi:10.1186/s12882-022-02719-5)
Supplement: Supplementary file 3 — Additional file 3. Simple linear regression analysis of hemodialysis patients. [file 12882_2022_2719_MOESM3_ESM.docx]

Simple linear regression analysis of hemodialysis patients.

|  | **Body structure and function** | | | | | | | | | **Activity** | | | **Participation** | | |  |  |
| --- | --- | --- | --- | --- | --- | --- | --- | --- | --- | --- | --- | --- | --- | --- | --- | --- | --- |
| **Hemodialysis patients** | **HS, Kgf** | | | **5STS, s** | | | **60sSTS, repetitions** | | | **SPPB, points** | | | **Participation scale, points** | | | | |
| **n=80** | β | p | r^2^ | β | p | r^2^ | β | p | r^2^ | β | p | r^2^ | β | p | r^2^ | |  |
| Age. *(yars)* | -0.184 | 0.102 | 0.034 | **0.348** | **0.003** | **0.121** | **-0.403** | **0.000** | **0.162** | **-0.437** | **0.000** | **0.191** | -0.115 | 0.312 | 0.013 | |  |
| Duration of HD (years*)* | -0.122 | 0.284 | 0.015 | 0.180 | 0.136 | 0.032 | -0.227 | 0.052 | 0.051 | -0.220 | 0.054 | 0.048 | -0.004 | 0.972 | 0.000 | |  |
| Diuresis Volume*(mL)* | 0.000 | 1.000 | 0.000 | -0.034 | 0.798 | 0.001 | 0.019 | 0.884 | 0.000 | 0.023 | 0.855 | 0.001 | -0.056 | 0.655 | 0.003 | |  |
| BMI *(kg/m²)* | - | - | - | - | - | - | - | - | - | - | - | - | -0.201 | 0.084 | 0.040 | |  |
| Body fat *(%)* | **-0.328** | **0.004** | **0.107** | 0.181 | 0.146 | 0.033 | - | - | - | **-0.345** | **0.003** | **0.119** | - | - | - | |  |
| ALM *(kg)* | **0.675** | **0.000** | **0.456** | -0.173 | 0.164 | 0.030 | 0.200 | 0.100 | 0.040 | **0.424** | **0.000** | **0.180** | -0.178 | 0.130 | 0.032 | |  |
| BMD total *(g/cm^3^)* | **0.509** | **0.000** | **0.260** | **-0.259** | **0.036** | **0.067** | **0.273** | **0.023** | **0.075** | **0.382** | **0.001** | **0.146** | - | - | - | |  |
| BMD spine *(g/cm^3^)* | **0.236** | **0.043** | **0.056** | - | - | - | - | - | - | - | - | - | - | - | - | |  |
| BMD hip (g/cm^3^) | **0.426** | **0.000** | **0.181** | -0.230 | 0.064 | 0.053 | **0.256** | **0.034** | **0.066** | **0.392** | **0.001** | **0.153** | -0.174 | 0.139 | 0.030 | |  |
| D vitamin *(ng/mL)* | **0.332** | **0.003** | **0.110** | -0.237 | 0.052 | 0.056 | 0.181 | 0.129 | 0.033 | **0.280** | **0.015** | **0.079** | -0.154 | 0.181 | 0.024 | |  |
| PTH *(pg/mL)* | 0.223 | 0.051 | 0.050 | **-0.314** | **0.009** | **0.098** | **0.283** | **0.016** | **0.080** | **0.341** | **0.003** | **0.116** | - | - | - | |  |
| Kt/V | **-0.284** | **0.011** | **0.081** | - | - | - | - | - | - | - | - | - | **0.241** | **0.032** | **0.058** | |  |
| Hb (*g/dL)* | **0.227** | **0.043** | **0.051** | - | - | - | - | - | - | - | - | - | - | - | - | |  |
| Alkaline phosphatase *(U/L)* | -0.197 | 0.080 | 0.039 | - | - | - | - | - | - | -0.215 | 0.059 | 0.046 | - | - | - | |  |
| Ferritin (*ng/mL)* | - | - | - | 0.174 | 0.150 | 0.030 | - | - | - | -0.184 | 0.110 | 0.034 | - | - | - | |  |
| HAP *(points)* | **0.370** | **0.001** | **0.137** | **-0.366** | **0.002** | **0.134** | **0.443** | **0.000** | **0.196** | **0.569** | **0.000** | **0.324** | **-0.241** | **0.036** | **0.058** | |  |
| BMI: body mass index; ALM: appendicular lean mass; BMD: bone mineral density; PTH: parathormone; CRP: C-reactive protein; Kt/V: fractional urea clearance; Hb: hemoglobin; HAP: human activity profile; HS: handgrip strength; 5STS: 5 repetitions sit-to-stand test; 60STS: 60 seconds sit-to-stand test; SPPB: Short Physical Performance Battery. | | | | | | | | | | | | | | | | |  |
